# Tumor-Derived Microvesicles Induce Proangiogenic Phenotype in Endothelial Cells via Endocytosis

**DOI:** 10.1371/journal.pone.0034045

**Published:** 2012-03-30

**Authors:** Taisuke Kawamoto, Noritaka Ohga, Kosuke Akiyama, Naoya Hirata, Shuji Kitahara, Nako Maishi, Takahiro Osawa, Kazuyuki Yamamoto, Miyako Kondoh, Masanobu Shindoh, Yasuhiro Hida, Kyoko Hida

**Affiliations:** 1 Department of Vascular Biology, Graduate School of Dental Medicine, University of Hokkaido, Sapporo, Hokkaido, Japan; 2 Department of Anatomy and Developmental Biology, School of Medicine, Tokyo Women's Medical University, Tokyo, Japan; 3 Department of Oral Pathology and Biology, Graduate School of Dental Medicine, University of Hokkaido, Sapporo, Hokkaido, Japan; 4 Department of Cardiovascular and Thoracic Surgery, Graduate School of Medicine, University of Hokkaido, Sapporo, Hokkaido, Japan; National Taiwan University Hospital, Taiwan

## Abstract

**Background:**

Increasing evidence indicates that tumor endothelial cells (TEC) differ from normal endothelial cells (NEC). Our previous reports also showed that TEC were different from NEC. For example, TEC have chromosomal abnormality and proangiogenic properties such as high motility and proliferative activity. However, the mechanism by which TEC acquire a specific character remains unclear. To investigate this mechanism, we focused on tumor-derived microvesicles (TMV). Recent studies have shown that TMV contain numerous types of bioactive molecules and affect normal stromal cells in the tumor microenvironment. However, most of the functional mechanisms of TMV remain unclear.

**Methodology/Principal Findings:**

Here we showed that TMV isolated from tumor cells were taken up by NEC through endocytosis. In addition, we found that TMV promoted random motility and tube formation through the activation of the phosphoinositide 3-kinase/Akt pathway in NEC. Moreover, the effects induced by TMV were inhibited by the endocytosis inhibitor dynasore. Our results indicate that TMV could confer proangiogenic properties to NEC partly via endocytosis.

**Conclusion:**

We for the first time showed that endocytosis of TMV contributes to tumor angiogenesis. These findings offer new insights into cancer therapies and the crosstalk between tumor and endothelial cells mediated by TMV in the tumor microenvironment.

## Introduction

Tumor blood vessels have been recognized as an important target for cancer therapy since Folkman introduced the concept that tumor growth is dependent on angiogenesis. Tumor angiogenesis is necessary for solid tumor progression and metastasis [1]. Tumor blood vessels provide nutrition and oxygen, promote tumor progression, and serve as gatekeepers for tumor cells to metastasize to other organs. Therefore, inhibition of tumor angiogenesis is a promising strategy for the treatment of cancer.

Tumor blood vessels are different from their normal counterparts and have altered morphology, altered blood flow, enhanced leakiness, structural abnormalities in the basement membrane, and abnormal pericytes [2]. Furthermore, there are many differences at the molecular and functional levels between tumor endothelial cells (TEC) and normal EC (NEC) [3], [4]. At the molecular level, TEC have distinct gene expression profiles [5].

We reported that TEC are cytogenetically abnormal [6], [7]. In addition, TEC showed higher migration potential and proliferation than NEC [8]. These results demonstrated that TEC maintain distinct biological differences from NEC as observed in tumor blood vessels in vivo. Furthermore, we reported that TEC have proangiogenic phenotype. For examples, TEC expressed high levels of the angiogenesis-related genes vascular endothelial growth factor (VEGF) and cyclooxygenenase-2 [9], [10]. However, the mechanism of acquisition of TEC abnormality has not been completely understood.

Recent studies report that crosstalk between tumor cells and stromal cells are important for tumor progression or acceleration of tumor malignancy by secreting microvesicles (MVs) [11], [12]. MVs have recently attracted attention among researchers of immune response and embryo development, playing a role in cell-to-cell communication [13]. MVs are released from various tumor cells and some progenitors of differentiated cells [14]. Tumor-derived MVs (TMV) contain bioactive molecules such as microRNAs (miRNA), mRNAs, and/or proteins inside themselves [15]. TMV are considered to facilitate extracellular matrix invasion and immune response evasion [16], [17], [18]. A few studies have shown TMV uptake by normal cells [14], [19]. However, the relationship between TMV uptake and its functions has not been elucidated, and it remains unclear how TEC are affected by TMV.

In the present study, to investigate the contribution for the specific phenotype of TEC, we analyzed the effect of TMV on NEC and the mechanism of their uptake.

## Materials and Methods

### Cell lines and culture conditions

A375-SM, a super-metastatic human malignant melanoma cell line, was kindly gifted by Dr. Isaiah J. Fidler (M.D. Anderson Cancer Center, Houston, TX). The cells were cultured in Minimum Essential Medium (GIBCO, Grand Island, NY) and supplemented with 10% fetal bovine serum. NEC were isolated according to the following method and cultured in a microvascular endothelial growth medium (Lonza, Basel, Switzerland) in a humidified atmosphere of 5% CO_2_ and 95% air at 37°C. Human microvascular endothelial cells (HMVEC) were purchased (Lonza, Basel, Switzerland) and cultured in microvascular endothelial growth medium (Lonza, Basel, Switzerland). Human renal epithelial cells (hREC) were purchased (Lonza, Basel, Switzerland) and cultured in renal epithelial cell growth medium (Lonza, Basel, Switzerland).

### Isolation of NEC

NEC were isolated from mouse dermal tissue using a magnetic activated cell sorting system (Miltenyi Biotec, Tokyo, Japan) with anti-CD31 antibody as previously described [6]–[10]. All animal experiments were performed following the regulation on animal experimentation of Hokkaido University. This study was approved by the Animal Care and Use Committee of Hokkaido University (approval ID: 08-0296). The binding of *Bandeiraea simplicifolia* isolectin B4 (BS1-B4 lectin) and the expression of CD31, CD105, and CD144 indicated a high purity of isolated EC in flow cytometric analysis. Furthermore, RT-PCR revealed that NEC expressed CD31, CD105, CD144, VEGF receptor-1, and VEGF receptor-2 (VEGFR1 and VEGFR2), indicating that NEC possessed EC characteristics during culture. Isolated EC were negative for the monocyte marker CD11b and hematopoietic marker CD45 by RT-PCR. Template-free samples were used as the negative controls.

### RT-PCR

Total RNA was extracted using the RNeasy Micro Kit (Qiagen, Valencia, CA, USA), and complementary DNA (cDNA) was synthesized using ReverTra-Plus (Toyobo, Osaka, Japan) as described previously [6]. cDNA was amplified by PCR and products were visualized by ethidium bromide staining under ultraviolet transillumination.

### Antibodies and reagents

Rat anti-mouse CD31 antibody and fluorescein isothiocyanate (FITC)-conjugated anti-mouse CD31 antibody was purchased from eBioscience (San Diego, CA, USA). FITC-conjugated BS1-B4 lectin was purchased from Vector Laboratories Burlingame, CA, USA). PE-conjugated anti-mouse CD31 antibody, anti-mouse CD105 antibody, rat anti-mouse CD144 antibody, and normal rat IgG were purchased from BD Pharmingen (San Diego, CA, USA). Anti-β-actin, total Akt, phospho (Ser473)-Akt, and anti-rabbit HRP-congujated secondary antibody were obtained from Cell Signaling Technology (Boston, MA, USA). LY294002 was obtained from Calbiochem (San Diego, CA, USA) and dynasore from Santa Cruz Biotechnology (Santa Cruz, CA, USA).

### Isolation of TMV

TMV were obtained from A375-SM as previously described [20]. Before isolation of TMV, FBS was ultracentrifuged at 110,000 *g* for 1 h. Then, A375-SM cultured media was replaced minimum essential medium supplemented with ultracentrifuged 10% FBS. After incubation for 2 days, the conditioned medium was collected and centrifuged at 2,000 *g* for 15 min at 4°C to thoroughly remove cellular debris. The supernatant was centrifuged again at 110,000 *g* for 70 min at 4°C. The pellets were washed with phosphate-buffered saline, and after ultracentrifugation, were resuspended in phosphate-buffered saline. Protein levels were determined using the BCA assay from Pierce Chemical Co (Rockford, IL, USA).

### Flow cytometry

For surface phosphatidylserine detection, TMV were resuspended in PBS and incubated with annexin V-FITC (Roche, Indianapolis, IN, USA) and anti-HLA-FITC for 30 min at room temperature. After washing to remove unbound reagents, size calibration of TMV was performed using microbeads (Invitrogen, Carlsbad, CA, USA) Next, TMV were analyzed on a BD FACS Aria II Flow Cytometer (BD, NJ, USA). Data were analyzed by Flow Jo (Tree Star, San Carlos, OR, USA).

### Scanning Electron Microscopy (SEM)

TMV were incubated on a coverslip at 37°C for 4 h. It was fixed with 2% glutaraldehyde in PBS. TMV were post fixed for 1 h with 1% osmium tetraoxide in PBS and were dehydrated in graded ethanol solutions of 50%, 70%, 95%, and 100% before being placed in an HCP-1 critical point drying machine (Hitachi, Tokyo, Japan). TMV were sputter-coated with platinum and palladium before being observed under an S4000 scanning electron microscope (Hitachi, Tokyo, Japan).

### Transmission Electron Microscopy (TEM)

TEM assay was performed as described previously [21]. After incubation with TMV (50 µg/ml) for 60 min, NEC were immersed in 2% glutaraldehyde in 0.1 M phosphate buffer for at least 2 h. The cells were treated with 1% OsO_4_ solution, dehydrated with a graded series of ethanol and propylene oxide, and embedded in epoxy resin. Ultra-thin sections (thickness, 60 nm) were cut, stained with lead citrate, and examined using HITACHI H-7000 electron microscope (HITACHI, Tokyo, Japan).

### TMV uptake assay

TMV were labeled using the PKH26 red fluorescent labeling Kit (Sigma-Aldrich, St. Louis, MO, USA) according to the manufacturer's instructions. Labeled TMV (50 µg/ml) were incubated at 37°C for 1 and 24 h. For FACS analysis, the cells were washed twice and trypsinized. After washing, the cells were resuspended in PBS containing 2 mM EDTA and 2% FBS. Next, PKH26 fluorescence in the cells was analyzed by FACS.

To determine PKH26-labeled TMV localization in NEC, the cells were fixed with 4% paraformaldehyde, washed, and stained with MFP488-phalloidin (MoBiTec, Göttingen, Germany) and 4′,6-diamidino-2-phenylindole (Roche, Indianapolis, IN, USA). PKH26-labeled TMV localization by ECs was determined using an FV10i confocal laser scanning microscope (Olympus, Tokyo, Japan).

### Random motility assay

Cells were plated on glass bottom dishes coated with poly-L-lysine (Matsunami, Osaka, Japan). After overnight incubation, the medium was changed with or without dynasore (50 µM) and LY294002 (80 µM) for 30 min. Cell movements were monitored with or without TMV (50 µg/ml) under an FV10i microscope (Olympus, Tokyo, Japan) at 10 min intervals. Velocity was determined by tracking the positions of cell nuclei using ImageJ (NIH, Bethesda, MD, USA). Migrated distance was integrated and divided by monitored time (12 h).

### Tube formation assay

Diluted Matrigel (BD Biosciences, San Jose, CA, USA) was transferred to a glass bottom dish and incubated at 37°C for 30 min to allow the matrix solution to solidify. Cells were harvested and resuspended in appropriate media and preincubated with or without dynasore (50 µM) and LY294002 (80 µM) for 30 min. Cells were then seeded at a density of 1×10^5^ cells per well with or without TMV (50 µg/ml), followed by incubation at 37°C for 12 h. Tube formation was observed using an inverted microscope and the experimental results were recorded at different times. The mean tube length was calculated using ImageJ.

### Western blotting

Western blot analysis was performed using antibodies specific to phosphorylated-Akt, total-Akt and HRP-conjugated secondary antibody as described previously [22]. Cells and TMV were lysed in RIPA buffer [50 mM Tris-HCl (pH = 7.4), 150 mM NaCl, 1 mM EDTA, 1% NP-40, 0.1% SDS, 0.5% sodium deoxycholate, and 1 mM Na_3_VO_4_] for 10 min on ice, and the lysate was clarified by centrifugation. Samples were electrophoresed on 10% polyacrylamide gels and transferred to Immobilon-P membranes (Millipore, Bradford, MA). The membranes were blocked for 60 min in TBS containing 5% skimmed dry milk and 0.1% Tween 20, and then incubated for overnight at 4°C with primary antibodies followed by incubation with HRP-conjugated secondly antibodies. Signals were developed using the ECL Western Blotting Detection Reagent (GE Healthcare, Little Chalfont, UK) and detected using an LAS-4000 mini image analyzer (FUJIFILM, Tokyo, Japan).

### Statistical analysis

Mean ±standard error (SE) values were compared using one-way ANOVA with Tukey-Kramer multiple comparison testing. When only two groups were compared, a two-sided Student's *t*-test was used.

## Results

### Isolation and culture of NEC

**Figure 1:**
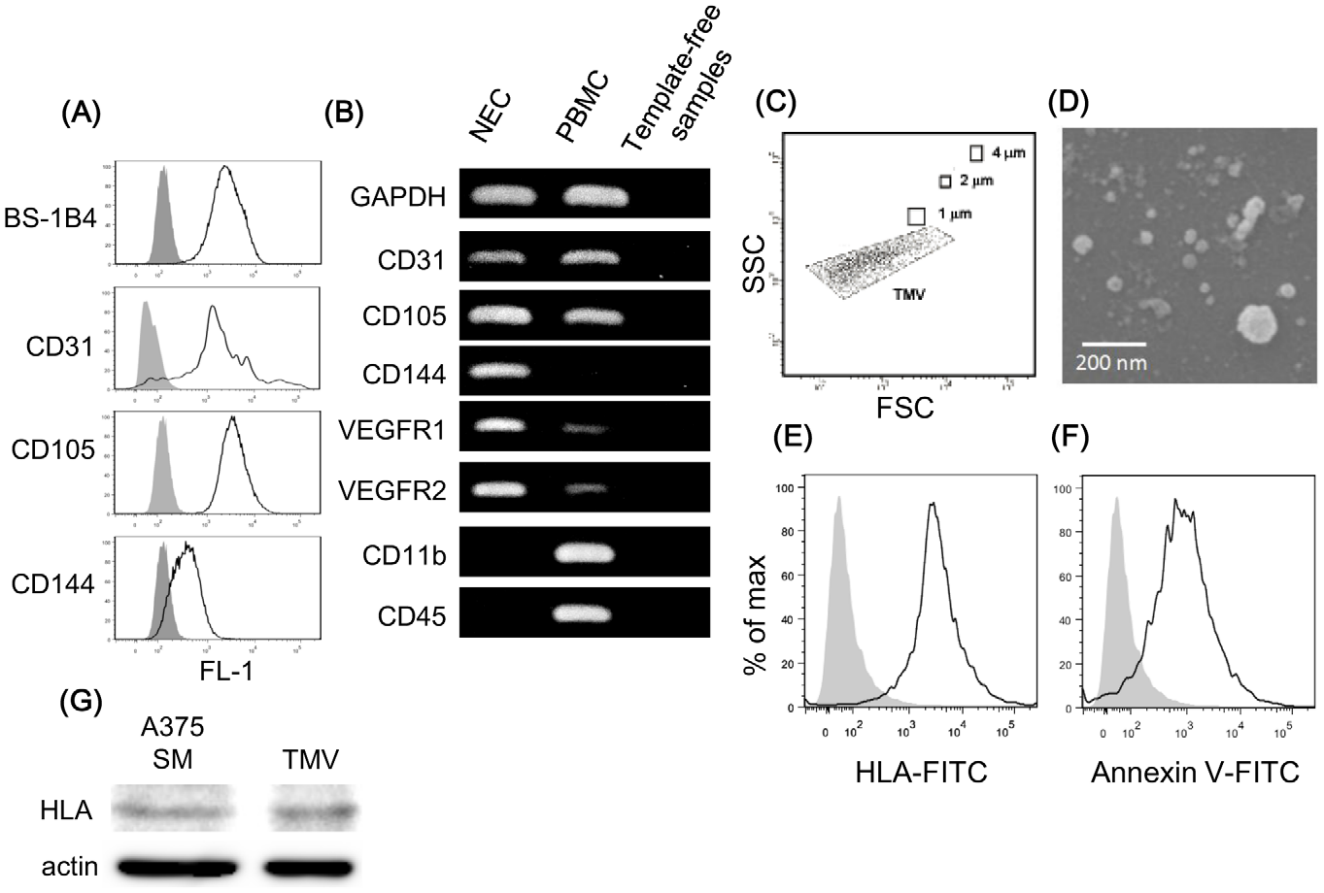
Characterization of isolated normal endothelial cells (NEC) from mice. (A) Representative flow cytometric analysis of NEC showing the expression of the endothelial markers CD31, CD105, CD144, and BS1-B4 lectin (mouse endothelial marker) (white area). Control levels with normal isotype IgG are shown in the gray area. Binding of BS1-B4 lectin and the expression of CD31, CD105, and CD144 revealed the high purity of the isolated NEC by FACS. (B) RT-PCR revealed that NEC expressed CD31, CD105, CD144, vascular endothelial growth factor receptor-1, 2 (VEGFR1 and VEGFR2) and not the monocyte marker CD11b and the hematopoietic marker CD45. To demonstrate that NEC were purified without any blood cell contamination, we also performed RT-PCR in mouse peripheral blood mononuclear cells (PBMC). Negative control was performed with template-free samples. (C) A375-SM cultured medium are analyzed by flow cytometry. The figure shows the forward and side scatter plot of the medium. A375-SM cells were cultured for 48 h. (D) Isolated TMV were observed by electron scanning microscopy. For sample preparation, TMV were coated on a glass cover for 4 h. (E, F) Flow cytometric analysis of TMV showing expression of HLA and phosphatidylserine (white area). (G) Western blotting analysis of TMV showing expression of HLA and β-actin. Same amount of proteins were loaded. These results indicate that some TMV might be shedding microvesicles derived from tumor plasma membrane.

NEC were isolated from mouse dermal tissue using a magnetic cell sorting system as described above. Flow cytometric analysis revealed the binding of BS1-B4 lectin and the expression of CD31, CD105, and CD144 in NEC (Figure 1A). RT-PCR revealed that NEC expressed CD31, CD105, CD144, VEGFR1, and VEGFR2, indicating that EC characteristics were present even during culture. Isolated EC were negative for the monocyte marker CD11b and hematopoietic marker CD45 by RT-PCR. As the negative controls, template-free samples were used (Figure 1B). These results showed a high purity of isolated NEC.

### TMV characterization

It has not been established conclusively how TEC acquire their abnormality in a tumor microenvironment. There are several reports that MVs secreted from malignant tumor cells (tumor-derived MVs: TMV) affect surrounding normal cells [23], [24]. In addition, we have found that a tumor-conditioned medium (tumor CM) induced some phenotypic change in such as enhanced motility (Figure S1A) and proliferation (Figure S1B). Furthermore tumor CM inhibited apoptosis induced by serum starvation in NEC (Figure S1C). Thus, we speculated that TMV contained in tumor-conditioned medium may cause phenotypic changes in ECs. To investigate how TMV contribute to the phenotypic change in NEC, we analyzed the effect of TMV secreted from a high-metastatic melanoma cell line, A375-SM cells.

TMV reportedly have a size of less than a few micrometers. Flow cytometric analysis of human TMV was performed to determine the size of TMV. Most isolated TMV were found to be around the forward and side scatter signal corresponding to 1-µm beads by flow cytometry, using micro beads as internal size standards (Figure 1C).

Scanning electron microscopy showed that TMV had a spheroid morphology less than 1 µm similar to flow cytometric assay (Figure 1D), although they were heterogenous in size.

Various reports have suggested that the surface of TMV express various cell membrane components, such as HLA originated from host cells [25] and phosphatidylserine [26], which consist of the inner leaflet of the cell membrane.

Isolated TMV were analyzed by flow cytometry using annexin V, which binds to phosphatidylserine, and anti-human HLA. These results suggested that TMV expressed phosphatidylserine and HLA (Figure 1E and 1F). Furthermore, western blotting analysis revealed TMV contains microvesicles markers, β-actin [27] and HLA (Figure 1G).

### TMV were taken up by NEC

A previous report indicates the possibility that protein and/or nucleic acids (mRNAs, miRNA) are packaged into MVs and can be taken up by cells, acting as biomolecules [14], [20], [28], [29]. To elucidate how TMV are taken up by NEC, TMV-treated NEC were analyzed by flow cytometry and confocal laser scanning microscopy.

**Figure 2:**
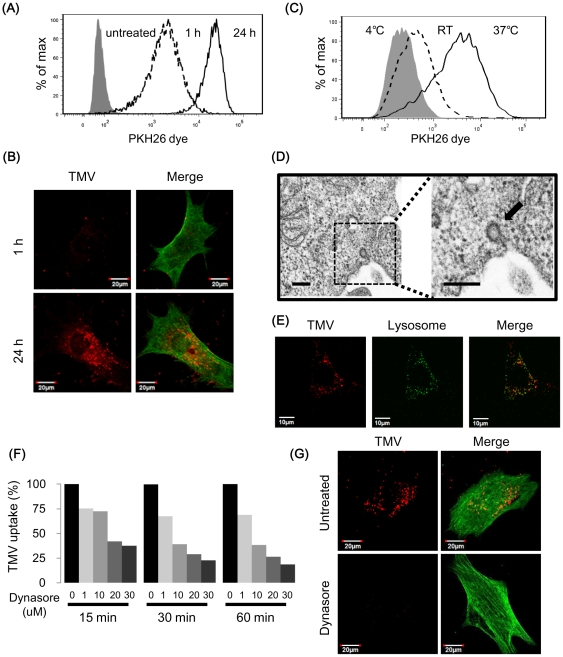
NEC take up TMV into cytoplasm depending on endocytosis. (A) Fluorescent intensity of TMV-treated NEC were measured by flow cytometry. Before treatment, TMV were stained with PKH26 dye. A dashed histogram shows NEC treated with TMV for 1 hr and an open histogram shows NEC treated with TMV for 24 h. (B) Fluorescent images of NEC treated with TMV for 1 and 24 h are shown. Green: F-actin, red: PKH26 -labeled TMV. Bar = 20 µm. (C) Flow cytometric analysis of TMV-treated NEC under low temperature. Filled histogram: 4°C, dashed histogram: room temperature, opened histogram: 37°C. NEC were pretreated in each temperature for 30 min and treated with PKH26 -labeled TMV for 30 min under each temperature. (D) The cells were incubated with TMV (50 µg/ml) for 60 min and fixed. The TEM images revealed that small particles were colocalized at engulfed plasma membrane in TMV-treated NEC (arrow). It was not observed in untreated-control. Bar = 2 µm. (E) TMV localization after endocytosis in NEC. NEC were stained with LysoTrakcer, fluorescent lysosome probe. Green: lysosome, red: PKH26 -labeled TMV. Bar = 10 µm. This result suggests that TMV were taken up via endocytosis. Endocytosis inhibitor blocked TMV uptake. (F) NEC were incubated with or without dynasore (1, 10, 20, 30 µM) for 30 min before treatment with PKH26-labeled TMV. After treatment with TMV for 15, 30, and 60 min, fluorescent intensity were measured by flow cytometry. TMV uptake ratio were compared with dynasore-treated NEC with untreated NEC. (G) Fluorescent images of NEC with or without dynasore (20 µM) are shown. Green: F-actin, red: PKH26 -labeled TMV. Bar = 20 µm.

After treatment with PKH26-labeled TMV for 1 and 24 h, NEC were analyzed for their fluorescent intensity using flow cytometry (Figure 2A). The fluorescent signal intensity was stronger in NEC after treatment with TMV for 24 h than after treatment for 1 h. Of note, confocal laser scanning microscopy analysis revealed that PKH26-labeled TMV were observed in cytoplasm after 1 and 24 h (Figure 2B). These results suggest that TMV were taken up by NEC.

Then, we hypothesized that TMV uptake is dependent on endocytosis because many types of extracellular fluids and nutrients are taken up by this process. To confirm this hypothesis, we performed the TMV uptake assay with low temperature treatment. Since endocytosis is energy-dependent, low temperature treatment is a common method for inhibition of endocytosis [30]. TMV uptake was inhibited at low temperature (Figure 2C), thus confirming that TMV uptake is endocytosis dependent. Furthermore, small particles were colocalized at engulfed plasma membrane by transmission electron microscopy (Figure 2D). These results suggested that TMV were taken up via endocytosis. In addition, staining NEC with LysoTracker revealed that some TMV are transported to the lysosome after endocytosis (Figure 2E).

Endocytosis requires a small GTPase dynamin, which pinches off endocytic vesicles from the cytomembrane [31]. Thus, we analyzed the effect of a specific dynamin inhibitor, dynasore, on TMV uptake. Flow cytometric analysis showed that dynasore inhibited TMV uptake in a dose-dependent manner (Figure 2F). TMV-treated NEC were observed by confocal laser scanning microscopy after treatment with/without dynasore. The resulting images are shown (Figure 2G).

These results suggested that TMV is taken up by NEC via endocytosis.

### TMV-induced proangiogenic property in NEC was inhibited by the endocytosis inihibitor

**Figure 3:**
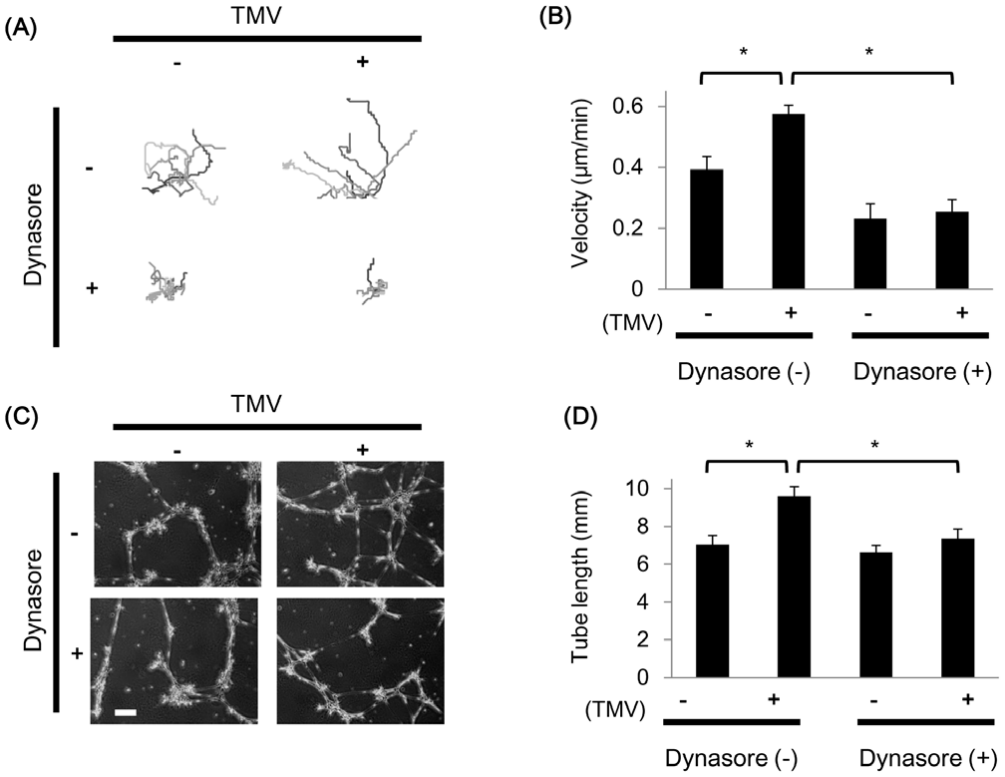
TMV promote random motility and tube formation in NEC. Time-lapse observation revealed that TMV promote random motility in NEC. When treated by the endocytosis inhibitor dynasore, cell motility was not stimulated anymore. (A) Migrated trajectories of TMV-treated NEC (with/without 50 µM dynasore) were plotted and velocity was calculated using ImageJ. (B) The results are presented as mean velocities ± SE (DMSO; n = 15, DMSO+TMV; n = 15, dynasore; n = 15, dynasore+TMV; n = 15). *P<0.01. TMV also promote tube formation in NEC. Dynasore canceled increase of tube formation even in NEC cultured with TMV. (C) Phase-contrast images of control and TMV-treated NEC (with/without 50 µM dynasore) cultured on a matrigel. Bar = 100 µm. Capillary-like structure enhanced with TMV. Total length of capillary-like tubes was analyzed using ImageJ. (D) The results are presented as mean tube length per field ± SE. (DMSO; n = 10, TMV; n = 10, dynasore; n = 10, dynasore+TMV; n = 10). *P<0.01.

We have shown that TEC have higher motility, which is important for angiogenesis, than NEC [8]. To analyze the effects of TMV on NEC, they were treated with TMV (50 µg/ml) for 12 h, and the random motility of cells was traced for 12 h using time-lapse videoscopy (Figure 3A).

Random motility of NEC increased significantly on TMV treatment compared with the control (*P*<0.01). When NEC were treated with the endocytosis inhibitor dynasore, TMV could not enhance NEC motility (Figure 3B).

Next, we investigated the effect of TMV on the angiogenic property of NEC by *in vitro* tube formation assay with/without dynasore. Representative data are shown (Figure 3C). The tube length of the capillary-like structure was calculated by ImageJ. After incubation with or without TMV (50 µg/ml) for 12 h, the total tube length was 1.37-fold longer in TMV-treated NEC than that in the control NEC (Figure 3D). However, dynasore suppressed TMV-stimulated tube formation. By apoptosis assay, dynasore (50 uM) did not show apoptotic changes in NEC (Figure S2). The inhibitory effects of dynasore on NEC were not caused by cytotoxicity. These results indicate that TMV uptake induces proangiogenic properties in NEC, at least in part via endocytosis.

### TMV activate the phosphoinositide 3-kinase (PI3K)/Akt pathway via endocytosis

**Figure 4:**
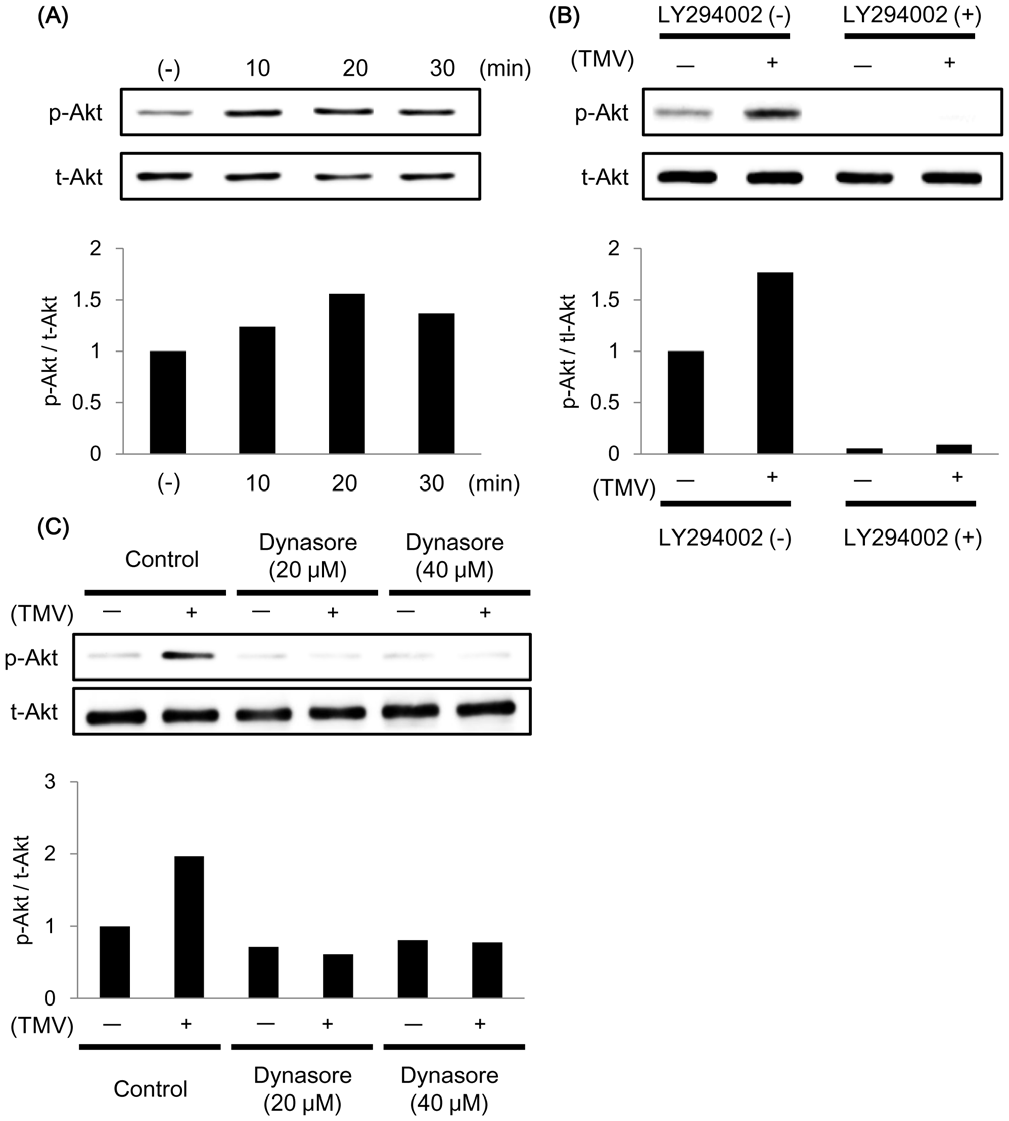
TMV activate the PI3K/Akt pathway. (A) TMV activated Akt in NEC. NEC were incubated with TMV (50 µg/ml) for the indicated time periods, and phosphorylated-Akt (p-Akt) and total-Akt (t-Akt) levels were determined in cell lysates by immunoblotting. Lower; Relative phosphorylated-Akt levels (p-Akt/t-Akt). (B) LY294002 inhibited Akt phosphorylation induced by TMV. NEC were preincubated with or without LY294002 (20 µM) for 2 h, then with or without TMV (50 µg/ml) for 20 min. Relative phosphorylated-Akt levels were quantified by immunoblotting. (C) The endocytosis inhibitor blocked Akt phosphorylation induced by TMV. NEC were preincubated with or without dynasore (20, 40 µM) for 30 min, and then with or without TMV (50 µg/ml) for 20 min. Relative phosphorylated-Akt levels were quantified by immunoblotting.

Akt phosphorylation is known to be involved in EC migration and tube formation [32]. From the results mentioned above, we hypothesized that TMV could activate Akt phosphorylation in NEC and analyzed the effect of TMV on Akt phosphorylation in NEC by western blotting. After treatment with TMV for 10 min, Akt phosphorylation was enhanced in NEC (Figure 4A). It is well known that Akt is regulated by PI3K, but recently, several reports suggest that Akt is also activated in a PI3K-independent manner [33].

Thus, we investigated whether TMV-induced Akt phosphorylation is dependent on PI3K. NEC were treated with LY294002 (20 µM) for 2 h, followed by stimulation with TMV. Akt phosphorylation was examined by western blotting. It was determined that LY294002 inhibited TMV-induced Akt phosphorylation (Figure 4B), which suggests that TMV activate the PI3K/Akt pathway in NEC.

Next, to investigate the activation of the PI3K/Akt pathway by TMV, we used dynasore. After NEC were pretreated with dynasore for 30 min, TMV no longer induced Akt phosphorylation (Figure 4C). These results indicate that TMV uptake via endocytosis results in activation of PI3K/Akt.

### TMV-induced proangiogenic phenotype is mediated by PI3K/Akt

**Figure 5:**
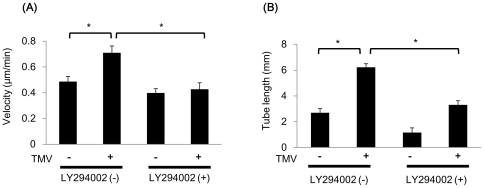
LY294002 inhibited TMV-promoted random motility and tube formation in NEC. (A) When treated by the PI3K inhibitor LY294002, cell motility was not stimulated by TMV (50 µg/ml) anymore. The results are presented as mean velocities ± SE (DMSO; n = 15, DMSO+TMV; n = 15, LY294002; n = 15, LY294002+TMV; n = 15). *P<0.01. (B) In addition, LY294002 suppressed increase of tube formation in NEC treated by TMV (50 µg/ml). The results are presented as mean tube length per field ± SE. (DMSO; n = 10, DMSO+TMV; n = 10, LY294002; n = 10, LY294002+TMV; n = 10). *P<0.01. These results indicate that TMV-induced proangiogenic phenotype is mediated by PI3K/Akt pathway, at least in part.

In order to analyze involvement of PI3K/Akt pathway in cell motility and tube formation enhanced by TMV, the PI3K inhibitor, LY294002 was used. TMV did not stimulate cell motility when NEC was treated with LY294002 (Figure 5A). Furthermore, LY294002 inhibited tube formation enhanced by TMV (Figure 5B). These results indicate that TMV induced proangiogenic phenotype on NEC through PI3K activation, at least in part.

## Discussion

The aim of this study was to investigate how TMV affect phenotypic changes in EC in a tumor microenvironment. This study yielded several results:

1. TMV are taken up by NEC via endocytosis.
2. TMV promote cell motility, tube formation, and Akt phosphoarylation in NEC.
3. TMV endocytosis is required for induction of the proangiogenic phenotype in EC.

In this study, we tried to isolate MV from normal cells as normal control. However, normal endothelial cells (HMVEC) and normal epithelial cells (hREC) secreted few amount of microvesicles compared to melanoma cells (Figure S3), consisting with the another report [34]. These results suggested that proangiogenic phenotypes as shown in this study are caused by TMV, not by MV from normal cells.

We previously reported that TEC had higher motility than NEC [8], and Akt phosphorylation levels in TEC were higher than those in NEC [35]. In addition, we observed that tumor-conditioned medium (tumor CM) induced enhanced cell growth or anti-apoptotic phenotype to NEC (Figure S1B, C). However, it remains unclear which factors contained in tumor-conditioned medium affect phenotypic changes in NEC. Despite this uncertainty, our results indicate that the uptake of TMV, which are contained in tumor-conditioned medium, induces phenotypic changes in NEC.

Although TMV are classified by size and several markers, their whole character have not been cleared, yet. In this study, isolated TMV had a wide range of size distribution observed by SEM and flow cytometry. TMV expressed HLA and β-actin, which are the markers for shedding microvesicle derived from tumor plasma membrane [36]. In addition, some TMV were smaller than 200 nm, suggesting that some of them might be exosomes. From these results, TMV have heterogeneous population. Other classifications are reported such as apoptotic bodies [19], microparticles [37], lipoprotein particles [38]. However, the correlation of size and biological function of TMV has not been elucidated.

There have been a few studies about the uptake on TMV by normal cells [14], [19]. The mechanism of the uptake of TMV has been controversial with regard to both endocytosis and membrane fusion [11]. In our study, the TEM revealed that small particles were colocalized at engulfed plasma membrane. It was not observed in untreated-NEC. In addition, TMV localized in the lysosome and dynasore inhibited the uptake of TMV by NEC. Thus, it was suggested that some membrane trafficking machinery, such as endosomal transport, may be used for TMV uptake [30]. These results showed that TMV are taken up via endocytosis. However, we did not investigated whether TMV are taken up via membrane fusion or not. Further study is needed.

Several reports have demonstrated that TMV can activate Akt phosphorylation [12], [39]. Recently, it was reported that the PI3K/Akt pathway affects EC motility and tube formation through the reorganization of actin cytoskeleton [40], [41]. We for the first time showed that cell motility and tube formation were enhanced via the PI3K/Akt pathway in normal cells by the uptake of TMV.

There have also been several reports that TMV transfer bioactive molecules to recipient cells [14], [20], [28], [29]. It is well known that receptor tyrosine kinase activation via growth factors regulates the PI3K/Akt pathway [42]. First, we analyzed the contents of TMV by cytokine array. However, there were minimal growth factors in TMV. This indicates that TMV-induced PI3K/Akt activation may not be caused by growth factors/receptor activation.

To investigate if TMV-induced motility of NEC is regulated in an autocrine manner, NEC was treated by TMV in bottom chamber of transwell. Migration assay was performed to analyze whether TMV-NEC can attract NEC from upper chamber in an autocrine manner or not (Figure S4A). As a result, the number of NEC which migrated toward the bottom chamber did not increase (Figure S4B). These results suggested that TMV-induced migration in NEC is not regulated in an autocrine manner.

Regarding with bioactive molecules in TMV, the possible bioactive molecules in TMV are phospholipids. Phosphatidylserine and sphingomyelin, which are the components of the membrane of TMV, activate Akt phosphorylation [43]. Moreover, other reports have indicated that the Ras-PI3K pathway is activated by endocytosis of virus micro particles [44]. Taken together, TMV endocytosis itself may also be important for activation of the signaling pathway in EC in tumor microenvironment.

In this study, we for the first time demonstrated that TMV are taken up by NEC via endocytosis. TMV uptake confers proangiogenic properties on NEC. Inhibiting TMV endocytosis may lead to the development of a useful prevention strategy against tumor angiogenesis.

## Supporting Information

Figure S1 **Tumor-conditioned medium activates NEC.** A375-SM cells were cultured in MEM (0.5% FBS supplemented). After two days, conditioned medium was centrifuged at 2,000 g to remove cells and debris. Random motility, cell proliferation and apoptosis were analyzed in NEC with NEC conditioned medium (NEC CM) or tumor conditioned medium (tumor CM). (A) Random motility was measured by time-lapse observation. Mean velocities are presented ± SE (NEC CM; n = 15, tumor CM; n = 15, p<0.01). Tumor CM enhanced motility in NEC. (B) NEC proliferation was measured using MTS assay kit (Promega, Tokyo, Japan). Mean MTS activity are plotted (n = 4). Cell proliferation was stimulated by Tumor CM. (C) The cells were stained with the Annexin-V-FLUOS Staining kit (Roche, Indianapolis, IN, USA) and were analyzed by flow cytometry (NEC-CM; n = 2, tumor CM; n = 4, bar = standard deviation, p<0.05). The percentages of dead cells decreased when NEC were treated by tumor CM.
(TIF)

Figure S2 **Analysis of cytotoxicity of Dynasore.** Cytotoxicity of dynasore was measured by apoptosis assay kit. NEC were treated with a various concentration of dynasore for 16 h. The cells were analyzed by flow cytometry. Dynasore was not cytotoxic to NEC at the concentration of 50 µM, that was used in our study.
(TIF)

Figure S3 **Normal cells secrete small amounts of MV.** A375-SM, HMVEC and hREC were seeded with 50% confluency. After two days, cells were trypsinized and total cell number were counted respectively and MV-derived from each cell lines were isolated as mentioned below. (A) Protein amount of each MV were measured by BCA protein assay kit. Then, secreted amount of MV per single cell were estimated. The amount of MV secreted by tumor and normal cell was analyzed. Under same conditions, the amounts of normal cell derived MV were little (HMVEC; 10 µg, hREC; 3 µg), compared with that of tumor cells (A375-SM; 435 µg). Secreted amount of MV per single cell (pg/cell) were presented. (B) MV numbers isolated from each conditioned medium was counted using flow cytometry. It was suggested that normal cell secreted little amount of MV. Counted numbers of MV in each conditioned medium are presented.
(TIF)

Figure S4 **TMV-induced migration was not regulated in an autocrine manner.** To discuss how TMV enhance motility in NEC, transwell assay (Corning, Tokyo, Japan) was performed according to manufacturer's protocol. (A) Briefly, NEC were seeded on bottom chamber (1×10^4^ cells/cm^2^). After 4 h, adhered NEC were treated with or without TMV (50 µg/ml) in bottom chamber. Then, NEC were seeded on upper chamber (1×10^5^ cells/ml). Twelve hours later, NEC were fixed with 10% formalin and stained with Mayer's Hematoxylin solution. (B) Migrated NEC numbers towards supernatant from NEC treated with or without TMV were counted (untreated; n = 12, with TMV; n = 12). NEC did not migrate towards supernatant from TMV-treated NEC in bottom chamber.
(TIF)
