# Supplementary figures and images for: Tumor-Derived Microvesicles Induce Proangiogenic Phenotype in Endothelial Cells via Endocytosis
Source: PLoS One. 2012 Mar 30;7(3):e34045. doi: 10.1371/journal.pone.0034045 (PMC3316594; doi:10.1371/journal.pone.0034045)

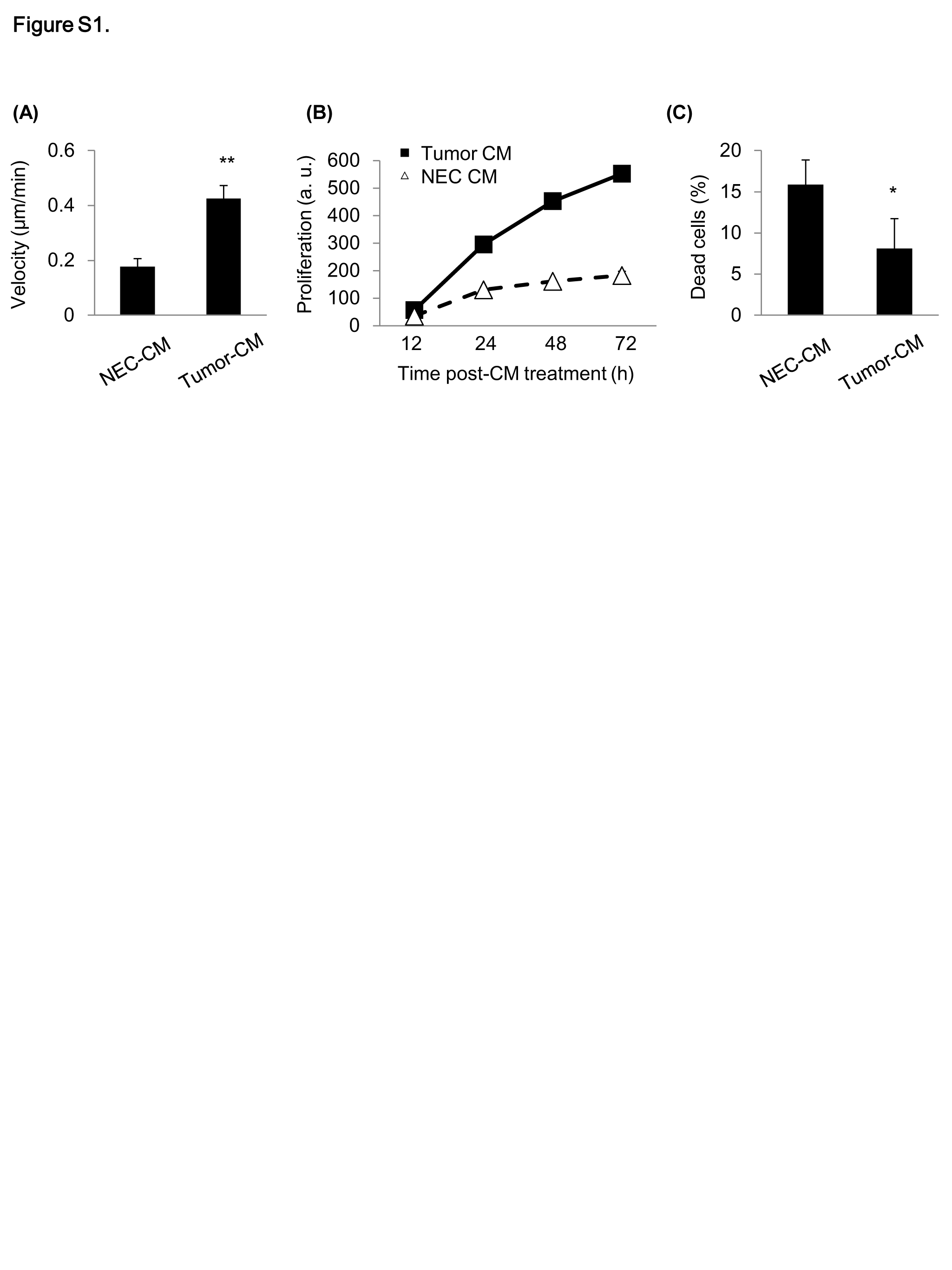

Supplement: Figure S1 — Tumor-conditioned medium activates NEC. A375-SM cells were cultured in MEM (0.5% FBS supplemented). After two days, conditioned medium was centrifuged at 2,000 g to remove cells and debris. Random motility, cell proliferation and apoptosis were analyzed in NEC with NEC conditioned medium (NEC CM) or tumor conditioned medium (tumor CM). (A) Random motility was measured by time-lapse observation. Mean velocities are presented ± SE (NEC CM; n = 15, tumor CM; n = 15, p<0.01). Tumor CM enhanced motility in NEC. (B) NEC proliferation was measured using MTS assay kit (Promega, Tokyo, Japan). Mean MTS activity are plotted (n = 4). Cell proliferation was stimulated by Tumor CM. (C) The cells were stained with the Annexin-V-FLUOS Staining kit (Roche, Indianapolis, IN, USA) and were analyzed by flow cytometry (NEC-CM; n = 2, tumor CM; n = 4, bar = standard deviation, p<0.05). The percentages of dead cells decreased when NEC were treated by tumor CM. (TIF) [file pone.0034045.s001.tif]

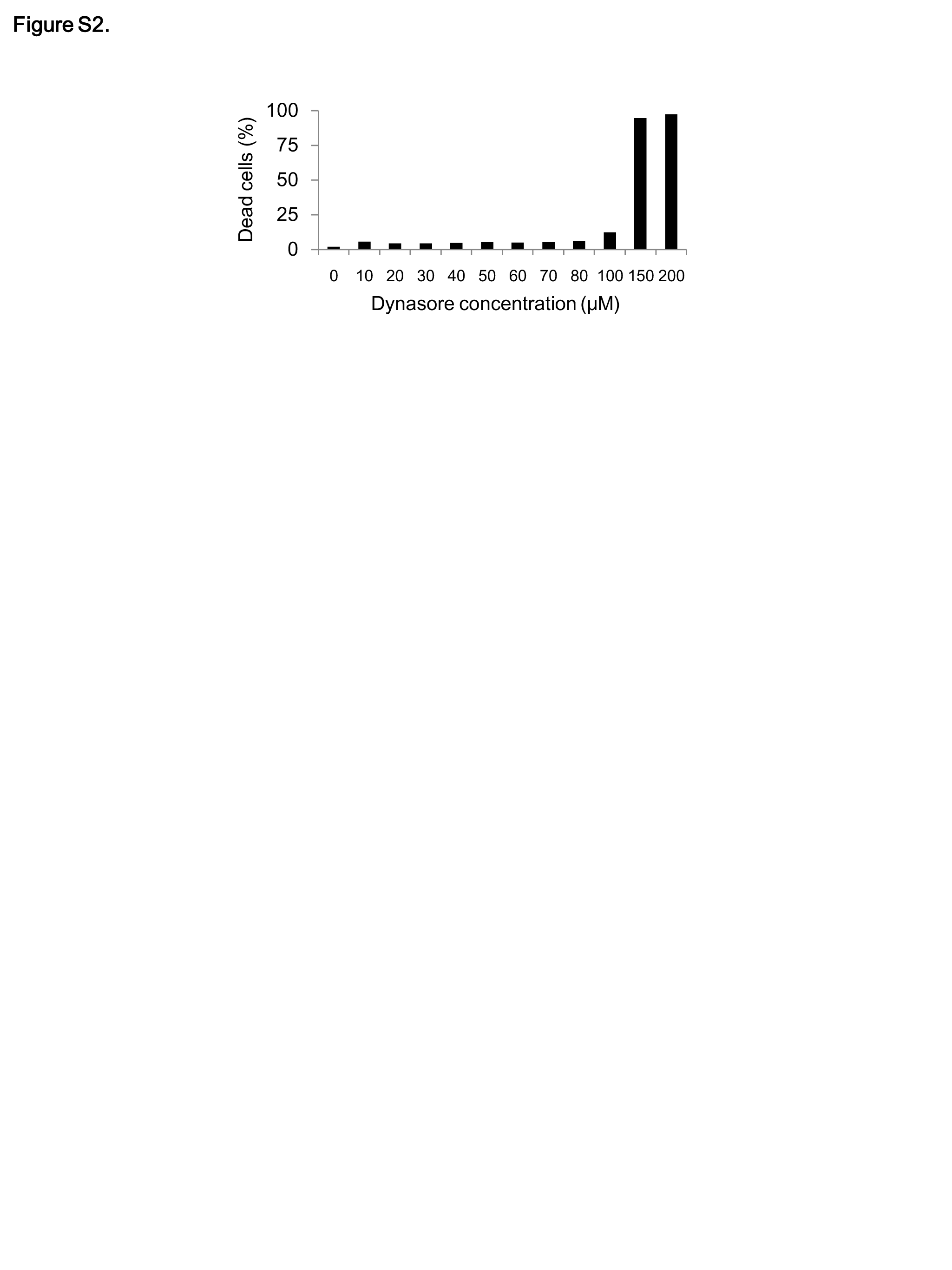

Supplement: Figure S2 — Analysis of cytotoxicity of Dynasore. Cytotoxicity of dynasore was measured by apoptosis assay kit. NEC were treated with a various concentration of dynasore for 16 h. The cells were analyzed by flow cytometry. Dynasore was not cytotoxic to NEC at the concentration of 50 µM, that was used in our study. (TIF) [file pone.0034045.s002.tif]

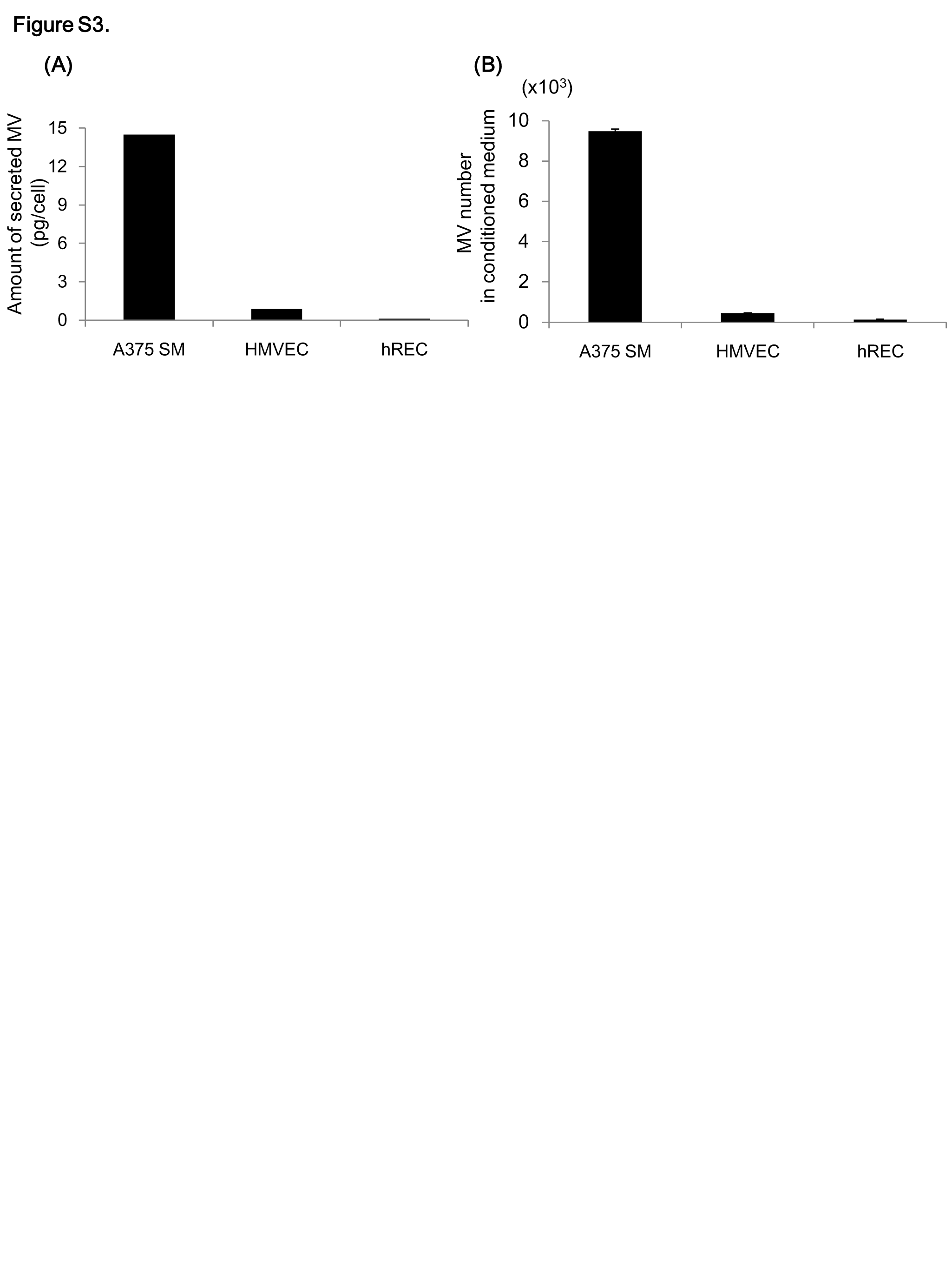

Supplement: Figure S3 — Normal cells secrete small amounts of MV. A375-SM, HMVEC and hREC were seeded with 50% confluency. After two days, cells were trypsinized and total cell number were counted respectively and MV-derived from each cell lines were isolated as mentioned below. (A) Protein amount of each MV were measured by BCA protein assay kit. Then, secreted amount of MV per single cell were estimated. The amount of MV secreted by tumor and normal cell was analyzed. Under same conditions, the amounts of normal cell derived MV were little (HMVEC; 10 µg, hREC; 3 µg), compared with that of tumor cells (A375-SM; 435 µg). Secreted amount of MV per single cell (pg/cell) were presented. (B) MV numbers isolated from each conditioned medium was counted using flow cytometry. It was suggested that normal cell secreted little amount of MV. Counted numbers of MV in each conditioned medium are presented. (TIF) [file pone.0034045.s003.tif]

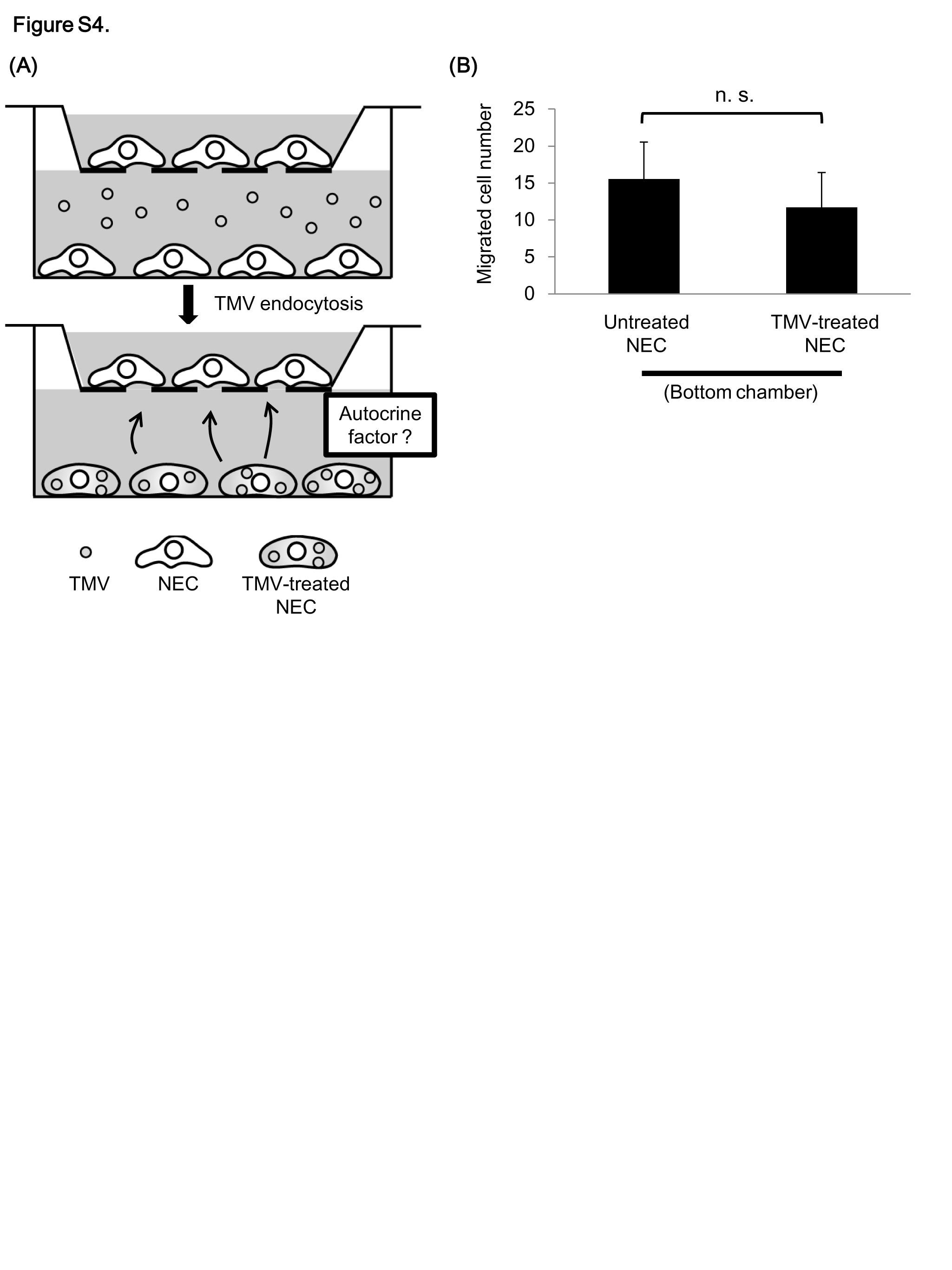

Supplement: Figure S4 — TMV-induced migration was not regulated in an autocrine manner. To discuss how TMV enhance motility in NEC, transwell assay (Corning, Tokyo, Japan) was performed according to manufacturer's protocol. (A) Briefly, NEC were seeded on bottom chamber (1×10^4^ cells/cm2). After 4 h, adhered NEC were treated with or without TMV (50 µg/ml) in bottom chamber. Then, NEC were seeded on upper chamber (1×10^5^ cells/ml). Twelve hours later, NEC were fixed with 10% formalin and stained with Mayer's Hematoxylin solution. (B) Migrated NEC numbers towards supernatant from NEC treated with or without TMV were counted (untreated; n = 12, with TMV; n = 12). NEC did not migrate towards supernatant from TMV-treated NEC in bottom chamber. (TIF) [file pone.0034045.s004.tif]
